# *Shank3* mutation manifests in abnormal gastrointestinal morphology and function in mice

**DOI:** 10.3389/fnins.2025.1552369

**Published:** 2025-04-17

**Authors:** Gari L. Eberly, Marie Manthey, Karen K. L. Pang, Heba Hussein, Emmanuel Vargas Paniagua, Scott Machen, Sara Maeve Klingensmith, Polina Anikeeva

**Affiliations:** ^1^MIT-Harvard Graduate Program in Health Sciences and Technology, Boston, MA, United States; ^2^K. Lisa Yang Brain-Body Center, Massachusetts Institute of Technology, Cambridge, MA, United States; ^3^Research Laboratory of Electronics, Massachusetts Institute of Technology, Cambridge, MA, United States; ^4^McGovern Institute for Brain Research, Massachusetts Institute of Technology, Cambridge, MA, United States; ^5^Department of Brain and Cognitive Sciences, Massachusetts Institute of Technology, Cambridge, MA, United States; ^6^Department of Electrical Engineering and Computer Science, Massachusetts Institute of Technology, Cambridge, MA, United States; ^7^Department of Biology, Wellesley College, Wellesley, MA, United States; ^8^Department of Materials Science and Engineering, Massachusetts Institute of Technology, Cambridge, MA, United States

**Keywords:** *Shank3*, enteric nervous system, gastrointestinal abnormalities, dysmotility, gastric permeability

## Abstract

**Background:**

Gastrointestinal (GI) comorbidities are common among those with Autism Spectrum Disorder (ASD), but their etiology is not well understood. This study aimed to characterize gastrointestinal morphology and function in Shank3B mutant mice, a common genetic model of ASD, to identify potential alterations to the GI tract that could underlie ASD-associated GI comorbidities.

**Methods:**

GI and enteric nervous system morphology was characterized using Hematoxylin and Eosin staining and immunohistochemistry. GI permeability was measured using the FITC-Dextran paracellular permeability assay. Whole-GI tract motility time was measured *in vivo* using the carmine dye motility assay. Colonic contractions were characterized by tracking motility using an *ex vivo* motility assay.

**Results:**

Homozygous knock-out (KO) *Shank3B^−/−^* mice exhibit significantly altered epithelial morphology and increased GI permeability. An increased myenteric plexus density and a higher number of HuC/D-expressing neurons in myenteric ganglia are observed in the colon of *Shank3B^−/−^* mice. These mice exhibit slowed whole-GI tract transit and reduced velocity and propagation length of colonic contractions. Compared to *Shank3B^−/−^* mice, heterozygous *Shank3B^+/−^* mice exhibit milder epithelial, neuronal, and functional alterations.

**Conclusion:**

*Shank3B^−/−^* mice exhibit altered GI morphology and function, while *Shank3B^+/−^* mice exhibit a partial phenotype. These results indicate that *Shank3,* whose mutation is associated with ASD, is critical for function of the GI tract and its mutation may contribute to the etiology of GI comorbidities.

## 1 Introduction

Autism Spectrum Disorders (ASD) are a group of heterogeneous neurodevelopmental disorders characterized by repetitive behaviors, hypersensitivity, and difficulty with communication (Leigh and Du, 2015; Balasco et al., 2020; Maenner, 2021). In the United States, approximately 1 in 36 children have been diagnosed with ASD; up to 80% of those diagnosed also experience gastrointestinal (GI) comorbidities including constipation and diarrhea (Adams et al., 2011; Bjørklund et al., 2020; Maenner, 2021; Deng et al., 2022). These comorbidities decrease quality of life and may exacerbate behavioral differences. The severity of GI symptoms is strongly correlated with the severity of behavioral challenges in ASD (Prosperi et al., 2017). Despite the prevalence of GI comorbidities, the relationship between ASD risk factors and altered GI function has not been fully elucidated.

GI function is locally regulated by the enteric nervous system (ENS), consisting of the submucosal plexus and myenteric plexus, that broadly control sensory and secretory or motility functions, respectively. In addition, the GI tract is extensively innervated by the efferent and afferent fibers that transduce motor and sensory signals from/to the central nervous system (CNS) (Rao and Gershon, 2016). In murine models, many ASD-linked genetic mutations have been shown to contribute to behavioral differences by altering central nervous system (CNS) development, but whether structural or functional changes in the GI tract and ENS are also present in these models is still being explored. Recent studies have found that ASD-linked mutations in genes including *Foxp*, *Cntnap2*, *Nlgn3*, and *SLC6A4* impact distinct aspects of myenteric plexus organization and function. Notably, distinct functional differences are observed even between models in which the same gene is mutated (Margolis et al., 2016; Fröhlich et al., 2019; Hosie et al., 2019; Leembruggen et al., 2020; Park et al., 2023; Robinson et al., 2023). These results suggest that the heterogeneous presentation of GI comorbidities in the ASD community is also represented in murine models, and that different ASD-linked mutations alter the ENS via distinct pathways. However, it is still unclear whether GI dysfunction associated with other ASD-linked mutations is in part due to altered GI tract development or secondary behavioral causes, such as altered diet and eating patterns (Hung and Margolis, 2023).

Haploinsufficiency of the *Shank3* gene manifests in Phelan-McDermid Syndrome (PMS), a monogenetic form of ASD (Phelan and McDermid, 2011; Tavassoli et al., 2021). Individuals with PMS present heterogeneously with developmental delay and intellectual disability, and some aspects of this heterogeneity have been linked to deletion size (Wilson, 2003). PMS is also associated with aberrations in GI secretions and motility (Malara et al., 2022; Matuleviciene et al., 2023). Gastroesophageal reflux is present in >40% of those with PMS, while chronic constipation and/or diarrhea is present in 26–57% of individuals with PMS (Soorya et al., 2013; Kolevzon et al., 2014; Sarasua et al., 2014; Matuleviciene et al., 2023). It is unknown whether the presence of GI comorbidities in PMS is also related to deletion size or variant. Due to the prevalence of GI symptoms in PMS, the impact of *Shank3* mutations on the intestinal microbiome, alongside histological characterization of the small intestine, has previously been studied in murine knockout (KO) models (Tabouy et al., 2018; Sauer et al., 2019; Delling and Boeckers, 2021; Wong et al., 2021).

In the CNS, SHANK3 is a postsynaptic density scaffolding protein that assists in dendritic spine formation in glutamatergic synapses (Schuetz et al., 2004; Raab et al., 2010; PubChem, 2024). SHANK3 has also been detected in neuronal cell bodies and nuclei, where it may impact protein transcription in response to synaptic activity (Grabrucker et al., 2014). SHANK3 is also found in GI epithelium, where it regulates tight junctions and zinc absorption (Raab et al., 2010; Pfaender et al., 2017; Sauer et al., 2019). *Shank3ΔC* KO (Δex21) mice exhibit more permeable intestinal barriers, a phenotype attributed to the role of *Shank3* as a regulator of tight junction protein ZO-1 expression (Wei et al., 2017). Altered epithelial morphology in the small intestine was observed alongside increased expression of ZO-1 and unaltered expression of tight junction protein Claudin3 in a different KO model, *Shank3αβ* (Δex11) (Sauer et al., 2019). While increased ZO-1 expression has been proposed as a marker for increased intestinal barrier permeability, intestinal barrier function remains to be directly measured *in vivo* in any *Shank3* KO model. Alterations in GI function have also been reported in zebrafish, where loss of *Shank3* led to decreased GI motility and lower numbers of serotonin-secreting enteroendocrine cells (James et al., 2019).

While these studies suggest that *Shank3* mutation could result in altered microbiome profiles and small intestine morphology, the impact of *Shank3* mutation on other aspects of GI organization or function remained to be reported. For instance, it was unknown whether *Shank3* mutation could lead to altered ENS organization, colon morphology, or GI motility in murine models. Furthermore, prior evaluations of intestinal barrier function in *Shank3* KO models relied on *ex vivo* assays that may disrupt tissue integrity and remove the contributions of extrinsic innervations, or did not directly measure paracellular permeability in GI tract. Overall, potential alterations in ENS organization, GI permeability, morphology, or motility within a given *Shank3* ASD murine model remain to be characterized.

Thus, in this study we sought to characterize the impact of *Shank3* mutation on ENS and GI tract morphology in the *Shank3B* (Δex13-16) mouse model, in which the homozygous KO (*Shank3B^−/−^*) exhibits more significant behavioral alterations compared to the heterozygous KO (*Shank3B^+/−^*) (Peça et al., 2011; Tzanoulinou et al., 2022). In the *Shank3B* model, exons 13–16 are deleted, resulting in the loss of the PDZ domain, which otherwise facilitates localization of postsynaptic density proteins (Monteiro and Feng, 2017). This deletion results in the complete loss of SHANK3_α_ and SHANK3_β_ isoforms, while some SHANK3_γ_ isoforms remain. Since humans with PMS are heterozygous for *Shank3* mutations (De Rubeis et al., 2018), in this study we characterize both homozygous and heterozygous KO mice.

Here, we first validate that the SHANK3 protein is present in the myenteric plexus, and that expression of SHANK3 is decreased in *Shank3B^−/−^* mice. We find that *Shank3B^−/−^* mice have significantly altered epithelial morphology and GI permeability. Furthermore, we find that adult *Shank3B^−/−^* mice exhibit increased myenteric plexus density in the distal colon as well as an increased number of neuron cell bodies in colonic myenteric ganglia. Whole-GI tract transit velocity is reduced in these mice. Further investigation via an *ex vivo* colonic motility assay reveals differences in contraction propagation distance and velocity. Heterozygous *Shank3B^+/−^* mice exhibit more mild phenotypic changes compared to *Shank3B^−/−^* mice, suggesting that this model is suitable for investigating severe and moderate GI dysfunction in ASD as well as for evaluating future therapeutic interventions.

## 2 Materials and methods

### 2.1 Animals

All experimental procedures were reviewed and approved by the MIT Committee on Animal Care (under protocol #2306000538). B6.129-Shank3^tm2Gfng^/J (RRID:IMSR_JAX:017688; JAX Strain #: 017688) mice (*Shank3B^+/−^* mice) were gifted by Dr. Guoping Feng (MIT) or obtained from the Jackson Laboratory and bred to obtain *Shank3B^−/−^*, *Shank3B^+/−^* and *Shank3B^+/+^* mice (Peça et al., 2011). Mice were kept in a 12:12 reverse light cycle and provided standard food and water *ad libitum*. Functional assays were performed during the dark cycle and at the same time of day (approximately zeitgeber time ZT15-16) to account for possible circadian influences on GI function (Paulose et al., 2019). Mice were housed by genotype following weaning to account for the possibility of behavioral differences impacting feeding behavior. Up to 5 adult mice were housed per cage. Mice were aged 8–12 weeks during experiments, and *Shank3B^+/+^* littermates were used as controls. Similar numbers of male and female mice were used in each experiment.

### 2.2 Histology

#### 2.2.1 Tissue preparation

To prepare intestinal cross-sections for Hematoxylin and Eosin (H&E) staining, mice were anesthetized under isoflurane and euthanized via cervical dissection (Bialkowska et al., 2016). The small intestine and colon were immediately removed, and luminal contents were flushed out with cold 4% PFA. The small intestine was cut into three equal segments (from proximal to distal: the duodenum, jejunum, and ileum) while the colon was kept intact. Mesenteric fat was removed from each segment; afterwards, each segment was cut open along the mesenteric border and pinned down mucosa-side up onto a dental wax-lined petri dish under light tension (CELLTREAT, Pepperell, MA, USA). Tissues were fixed with 4% PFA for 24 h at 4°C. After fixation, each segment was rolled into a “swiss roll” kept intact with an insect pin and stored in PBS at 4°C for at least 24 h before being processed to paraffin (Moolenbeek and Ruitenberg, 1981). Tissues were paraffin processed and embedded at the Koch Histology Core at MIT. For morphological analysis, tissue was sliced into 5 μm thick paraffin sections and stained with Hematoxylin and Eosin using an auto-stainer (Tissue-Tek Prisma, Sakura Finetek, USA). An Aperio Digital Slide scanner was used to obtain brightfield images (at 20X magnification) of tissue sections. Morphological measurements (including villi height and crypt depth) were obtained using the ImageScope x64 software (Leica Biosystems, Nussloch, Germary). Investigators were blinded to the genotype.

To prepare whole-mount tissue for immunohistochemistry, mice were deeply anesthetized with pentobarbital sodium (Fatal-Plus, Vortech Pharmaceuticals, Dearbon, MI, USA) and transcardially perfused with ice-cold PBS followed by ice-cold 4% PFA. The small intestine and colon were dissected, and images were taken to measure intestinal length. Colon length was manually measured using ImageJ/Fiji (Schindelin et al., 2012). Following the same process reported for swiss-roll processing, the tissue segments were cleaned, cut, and pinned. Tissues were fixed for 1 h in 4% PFA at 4°C followed by storage in PBS at 4°C. Tissues were stored while pinned under light tension. Prior to staining, 2–3 cm long samples were cut, and the muscle layer (containing the myenteric plexus) was peeled away from the mucosa using two pairs of tweezers.

#### 2.2.2 Immunohistochemistry

For whole-mount immunohistochemistry, samples were stained in a 24-well plate (CELLTREAT, Pepperell, MA, USA). All steps at room temperature were performed on a shaker. Samples were washed three times in PBS, then permeabilized in 1% PBS-T for 1 h followed by blocking in 3% donkey buffer serum (DBS) in 1% PBS-Triton (PBS-T) for 2 h. Samples were stained with unconjugated primary antibodies (Anti-Shank3: 1:250, Novus Biologicals, NBP1-46768) diluted in 5% DBS in 0.1% PBS-T overnight at 4°C then washed three times with 3% DBS in 0.1% PBS for 30 min each. Samples were stained with secondary antibodies (anti-rabbit IgG (H + L), Alexa Fluor 568: 1:1000, Invitrogen, A10042) for 2 h at room temperature, then washed three times with 3% DBS in 0.1% PBS-T for 30 min each. Next, samples were stained with conjugated primary antibodies (Anti-PGP 9.5 Alexa Fluor 488: 1:500, Abcam, ab302578; Anti-HuC/D Alexa Fluor 647: 1:500, Abcam, ab237235) overnight at 4°C followed by washing. Samples were subsequently stained with DAPI (1:20000, Invitrogen, D1306) for 30 min at room temperature, washed, then placed on a slide with an attached 0.5 mm spacer. Samples were then flattened with a miniature paintbrush and mounted with Fluoromount-G mounting medium (Invitrogen, Cat. No. 00–4,958-02).

### 2.3 Quantification

#### 2.3.1 Image acquisition

A Leica DMi8 confocal microscope (Leica Microsystems, Wetzlar, Germany) was used to obtain fluorescent images. Images were collected using a 20X objective (for plexus morphology and ganglionic cell count) or a 63X oil-immersion objective (for SHANK3 expression), resulting in total magnification of 200X or 630X, respectively. Z-stacks (up to approximately 50 μm thick) were acquired to capture the entire ganglionic structure in the image, and for larger areas of interest, tiled images were stitched together using the LASX software (Leica Microsystems, Wetzlar, Germany). Z-stacks were combined using maximum projection prior to analysis.

#### 2.3.2 Image analysis

##### 2.3.2.1 SHANK3 expression

Average SHANK3 fluorescence signal intensity per ganglionic region (as marked by PGP 9.5 expression) was found using Aivia Software 14 (Leica Microsystems, Wetzlar, Germany) and normalized to PGP 9.5 expression. ROIs were manually drawn around ganglia, after which the ganglionic area within the ROI was identified using the Cell Count function, and the fluorescent intensity associated with PGP 9.5 and SHANK3 in each ganglion was determined using the fluorescent intensity measurement tool. SHANK3 expression was measured in multiple ganglia per mouse, and the mean is reported.

##### 2.3.2.2 Plexus morphology

1,200 μm × 1,200 μm images of the myenteric plexus were taken for plexus density measurements. Images were analyzed using the REAVER MATLAB program (Corliss et al., 2020). Briefly, the image was blurred with an averaging filter to estimate background illumination, which was subsequently subtracted from the image. The background-subtracted image was lightly blurred, converted to grayscale, and thresholded to obtain an initial segmentation. Borders of the initial segmentation were then cleaned using an averaging filter, followed by a series of morphological operations to close holes. Given that connected regions of the plexus were generally >1,200 pixels in area, segmented regions less than this value were removed, as were regions with a segment diameter <8 pixels. The initial segmentation was checked by the user, and if necessary, manually edited for accuracy. Plexus density was calculated by dividing the number of pixels in the plexus segmentation by the total amount of pixels in the image.

Neuronal cell body count per ganglion was quantified using the Aivia Pixel Classifier. First, example neuron cell bodies, as marked by HuC/D expression, were manually segmented. This training set was used to train the pixel classifier, which was subsequentially used to segment and count the number of neurons per ganglia. ROIs were manually drawn around each ganglion, and pixel classification was performed in each ROI.

### 2.4 Functional assays

#### 2.4.1 Whole-GI transit

Whole GI transit time was determined using the previously described carmine dye assay (Koester et al., 2021). Mice were fasted for 1 h prior to the study. Briefly, at zeitgeber time ZT15-16, mice were orally gavaged with 150 μL of 6% carmine dye in 0.5% methyl cellulose solution in dH_2_O in a red-light illuminated room, then placed in individual cages with access to water and chow. Every 10 min, cages were checked for the presence of a dyed fecal pellet.

#### 2.4.2 *Ex-vivo* colonic motility assay

Mice were anesthetized under isoflurane and euthanized via cervical dissection. The entire length of the colon was dissected out. The colon was placed in ice-cold (4°C) Krebs buffer saturated with carbogen, (95% O_2_/5% CO_2_), the mesentery was carefully removed, and the luminal contents were flushed with a blunt needle and 5 mL syringe. The colon was then placed in an organ bath with a constant flow of warm Krebs solution (35°C, 3.5 mL/min) aerated with carbogen. Each colon was acclimated to the bath for 30 min prior to video recordings. Motility videos were recorded for 15 min. From these videos, spatiotemporal maps of colonic motility were created using the Gastrointestinal Motility Monitor (GIMM; Catamount Research and Development, St. Albans, VT, USA) (Hoffman et al., 2010).

Spatiotemporal maps were analyzed using a custom Matlab (The MathWorks Inc., Natick, MA, USA) program. Spatiotemporal map matrices (in which each column in the matrix corresponds to a point in time, and each row in the matrix represents a location along the colon) were imported into Matlab. Each row was normalized by the mean pixel intensity of the row to remove banding artifacts corresponding baseline differences in colon diameter. Next, the matrix was averaged using a gaussian filter (h = 8, sigma = 3) to smooth the contractile pattern. To find the number of initiated contractions, the absolute value of intensity profiles across time from approximately the first 5% of the proximal colon were plotted, where peaks in the intensity profile represented contraction events. Peaks were identified using findpeaks() function, and contraction events that were not completed by the end of the recording were discarded. Similar intensity peak profiles were utilized to find the spatiotemporal map coordinates that corresponded to the beginning and end of each contraction. Intensity profiles were generated for approximately every 10% of the intensity map. These profiles, and whether peaks associated with contractions could be detected within them, were used to localize the manual selection of the start and end time and position of each contraction. From these coordinates, the velocity and duration of each contraction was calculated, as was the inter-contraction interval.

#### 2.4.3 FITC-Dextran permeability assay

The FITC-Dextran permeability assay was conducted as previously described (Volynets et al., 2016; Woting and Blaut, 2018). Briefly, mice were fasted for 6 h (beginning at zeitgeber time ZT12), then gavaged with a PBS solution containing 80 mg/mL FITC-Dextran 4,000 kDa at a dose of 600 mg/kg body weight. FTIC-Dextran solutions were kept in the dark, on ice, prior to gavage. After 45 min, blood was collected from the submandibular bundle in heparinized tubes. To separate plasma, collected blood was centrifuged at 6000 rpm for 10 min at 5°C. Plasma was then collected and diluted 1:7 with PBS. Plasma fluorescence was measured using a SpectraMax M2 Microplate Reader (Molecular Devices) with an excitation wavelength of 485 nm and an emission wavelength of 515 nm. Sample fluorescence was measured three times per well, and the average is reported. Plasma fluorescence was determined by comparing measured values to a standard curve (FITC-Dextran dissolved in PBS).

### 2.5 Statistical analysis

Statistical analysis was performed in GraphPad Prism (version 10.2.0, for Windows, GraphPad Software, Boston, MA, USA, www.graphpad.com). Either a one-way ANOVA or a two-way ANOVA in combination with Tukey’s multiple comparisons tests (threshold *p* values = 0.05) was used to identify significant differences between genotypes and intestinal regions. Significant p value results from these tests are displayed on their respective graphs. Data are reported as mean ± SEM (standard error of the mean).

## 3 Results

**Figure 1:**
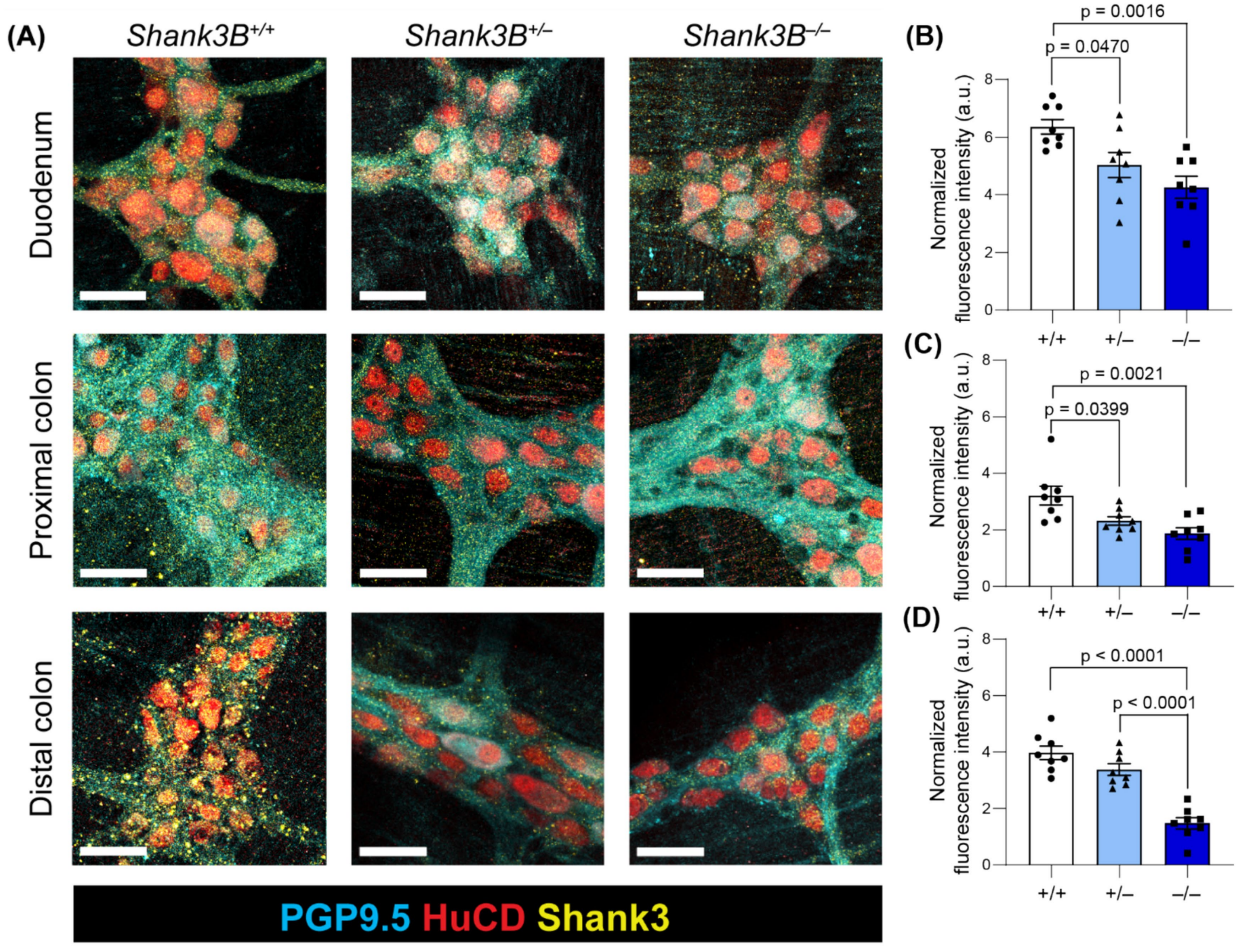
**(A)** Myenteric plexus SHANK3 expression in regions of the GI tract in *Shank3B*^+/+^, *Shank3B*^+/−^, and *Shank3B*^−/−^ mice. Myenteric neurons exhibit punctate expression of SHANK3 (yellow) in cell bodies (HuC/D – red) and projections (PGP 9.5 – cyan). SHANK3 expression is diminished in *Shank3B*^+/−^ and *Shank3B*^−/−^ mice. Scale bars indicate 40 μm. **(B–D)** Quantification of SHANK3 expression in myenteric ganglia in the duodenum **(B)**, proximal colon **(C)**, and distal colon **(D)** normalized by PGP 9.5 expression (*n* = 8). One-way ANOVA with Tukey’s multiple comparison test.

SHANK3 protein has previously been detected in GI epithelium lysate, and single-cell RNA sequencing has identified *Shank3* transcripts in some enteric neurons (Sauer et al., 2019; Drokhlyansky et al., 2020). To assess *Shank3* expression in enteric neurons, we employed immunostaining for SHANK3 protein in myenteric plexus ganglia in the duodenum, proximal colon, and distal colon. We found that SHANK3 is indeed expressed in the soma and axonal projections of myenteric neurons, and that SHANK3 expression is significantly reduced in the duodenum and proximal colon of *Shank3B^+/−^* and *Shank3B^−/−^* mice, as well as the distal colon of *Shank3B^−/−^* mice. In the distal colon of *Shank3B*^+/*−*^ mice the expression of SHANK3 trended lower compared to *Shank3B^+/+^ mice,* but this effect was not statistically significant (Figures 1A–D).

**Figure 2:**
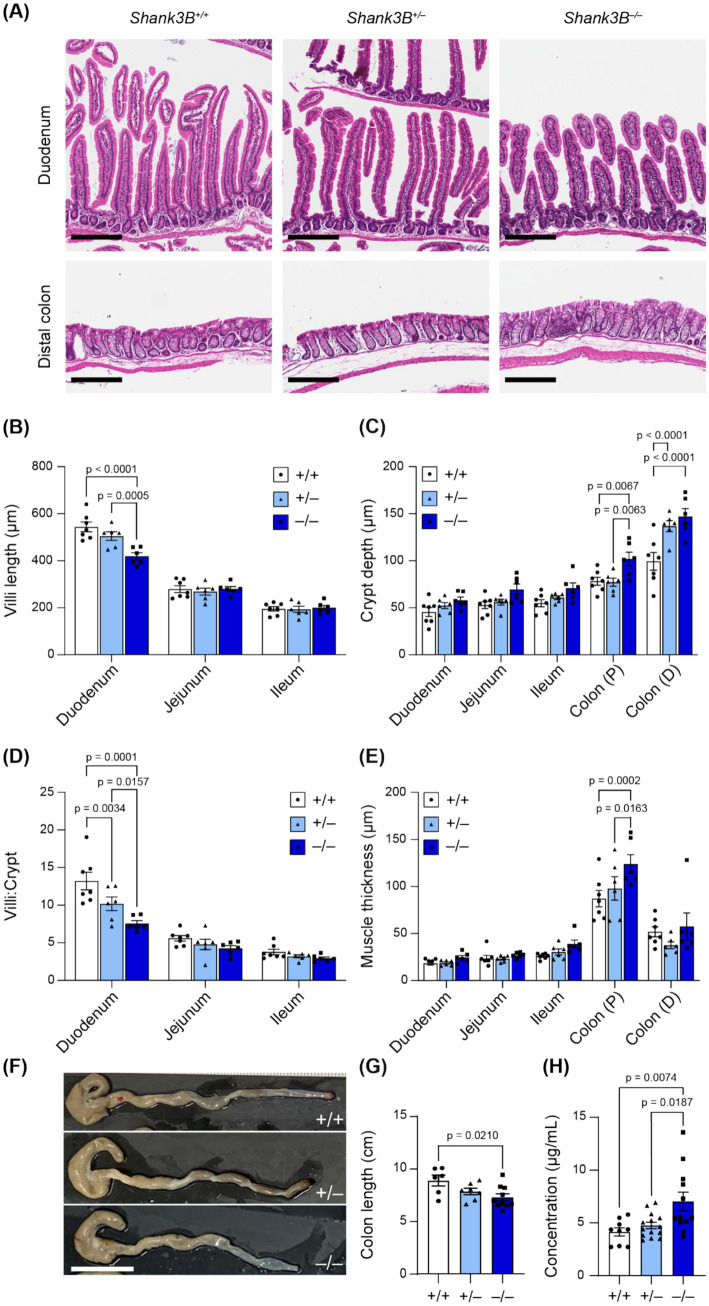
**(A)** Representative H&E cross-sections. Scale bars indicate 200 μm. **(B–E)** Morphological analysis of H&E cross-sections (*n* = 6–7). 10 measurements were taken per animal, and data reported as mean ± SEM, with individual data points representing the mean value from each animal. Two-way ANOVA with Tukey’s multiple comparison test. **(B)** Villi length. **(C)** Crypt depth. **(D)** Villi:crypt ratio. **(E)** Muscle thickness (longitudinal and circular). **(F)** *Shank3B*^−/−^ mice have shorter colons (*n* = 7–10). **(G)** Quantification of **F**. One-way ANOVA with Tukey’s multiple comparison test. **(H)** FITC-Dextran concentration in plasma is increased in *Shank3B*^−/−^ mice, indicative of higher GI epithelial permeability (*n* = 9–14). One-way ANOVA with Tukey’s multiple comparison test. Colon (P): Proximal colon. Colon (D): Distal colon.

The absorptive and digestive functions of the GI tract depend on the integrity of the GI mucosa, and altered GI morphology has previously been observed in individuals with inflammatory bowel disease as well as in other murine models of ASD (Erben et al., 2014; Langner et al., 2014; Wang et al., 2023). Given that SHANK3 is expressed in GI epithelium and mucosa, we asked whether loss of *Shank3* leads to alterations in gross GI tract morphology and tissue organization (Drokhlyansky et al., 2020). To determine whether tissue organization was altered, features including villi length, crypt depth, and muscle thickness were measured in sections of the small intestine and colon (Figures 2A–E). While gross organization remained intact between *Shank3B^+/+^*, *Shank3B^+/−^*, and *Shank3B^−/−^* mice, villi length was shorter in the proximal small intestine (duodenum) of *Shank3B^−/−^* mice (^−/−^: 418 ± 15 μm, *n* = 6; ^+/−^: 504 ± 18 μm, *n* = 6; ^+/+^: 543 ± 20 μm, *n* = 7) (Figure 2B). *Shank3B^−/−^* mice were also found to have larger crypts in the colon (Figure 2C). To test whether this difference in villi length was simply due to *Shank3B^−/−^* mice exhibiting shorter intestines, villi:crypt ratio was compared, and *Shank3B^−/−^* mice were found to have a significantly lower villi:crypt ratio compared to *Shank3B^+/+^* and *Shank3B^+/−^* mice (Figure 2D). Notably, heterozygous *Shank3B^+/−^* mice were also found to have a lower villi:crypt ratio than *Shank3B^+/+^* mice. Finally, *Shank3B^−/−^* mice exhibited a thicker muscle layer in the proximal colon (Figure 2E). *Shank3B^−/−^* mice were also found to possess shorter colon length as compared to *Shank3B^+/+^* and *Shank3B^+/−^* mice (^−/−^: 7.20 ± 0.33 cm, *n* = 10; ^+/−^: 7.75 ± 0.30 cm, *n* = 7; ^+/+^: 8.79 ± 0.52 cm, *n* = 6) (Figures 2F,G).

Given these alterations in epithelial organization and that *Shank3* modulates the expression of tight junction proteins in GI epithelium (Wei et al., 2017), we next evaluated whether loss of *Shank3* impacts intestinal barrier function. Epithelial barrier integrity is important for absorption regulation and blocks inflammatory luminal antigens from entering the body (Chelakkot et al., 2018). Increased epithelial barrier permeability has previously been reported in individuals with ASD, and is hypothesized to contribute to increased inflammation as well as pathological changes in metabolism (Arrieta et al., 2006; de Magistris et al., 2014). To assess epithelial barrier permeability, we orally gavaged fasted mice with a fluorescent solution of FITC-Dextran 4,000 kDa, and then quantified FITC-Dextran fluorescence in blood serum. We found a significantly higher concentration of FITC-Dextran in the blood serum of *Shank3B^−/−^* mice as compared to *Shank3B^+/+^* and *Shank3B^+/−^* littermates 45 min after gavage, indicative of higher epithelial barrier permeability (^−/−^: 7.03 ± 0.87 μg/mL, *n* = 12; ^+/−^: 4.75 ± 0.31 μg/mL, *n* = 14; ^+/+^: 4.15 ± 0.39 μg/mL, *n* = 9) (Figure 2H). This change in intestinal permeability, as well as the decreased surface area of the small intestine due to smaller villi, did not impact the weight of adult mice (Supplementary Figure S1).

**Figure 3:**
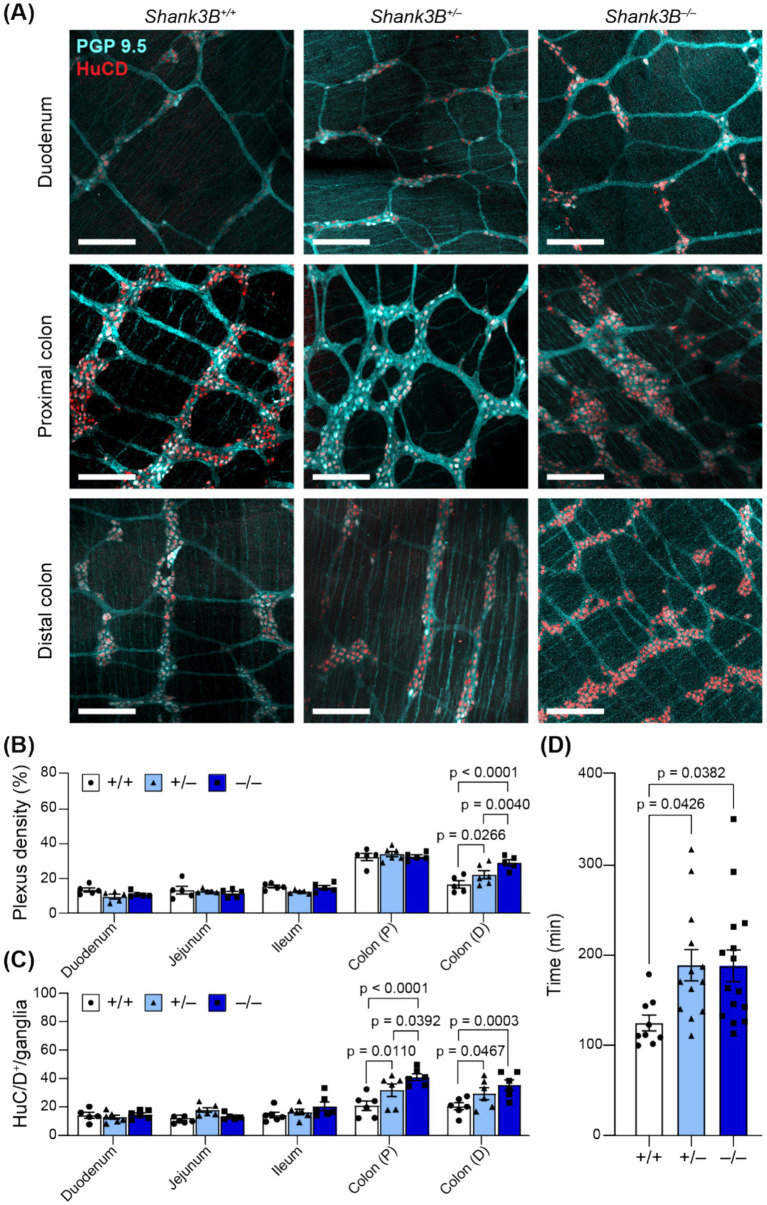
**(A)** Representative images of myenteric plexus morphology in regions of the GI tract in *Shank3B*^+/+^, *Shank3B*^+/−^, and *Shank3B*^−/−^ mice. Scale bars indicate 200 μm. Neuronal bodies indicated by HuC/D (red) and projections indicated by PGP 9.5 (cyan). **(B)** Plexus density is increased in the distal colon of *Shank3B*^−/−^ mice (*n* = 5–6). Two-way ANOVA with Tukey’s multiple comparison test. **(C)** Number of neurons per ganglia are decreased in the duodenum, and increased in the colon, as marked by HuC/D^+^ cells. Ganglia grouped by genotype (*n =* 5–6). **(D)** GI transit time is increased in *Shank3B*^−/−^ mice (*n* = 9–15). One-way ANOVA with Tukey’s multiple comparison test. Colon (P): Proximal colon. Colon (D): Distal colon.

Alterations in ENS organization (including decreased number of enteric neurons, and increased density of the myenteric plexus) have been linked to alterations in GI motility and have previously been reported in other murine models of ASD (Niesler et al., 2021; Wang et al., 2023). We found that myenteric plexus density was increased in the distal colon of *Shank3B^−/−^* mice (^−/−^: 28.9% ± 1.75%, *n* = 5; ^+/−^: 20.87 ± 2.49%, *n* = 5; ^+/+^: 16.49 ± 2.09%, *n* = 5; Figures 3A,B), which was also correlated with an increase in number of enteric neurons (as marked as HuC/D^+^) per ganglion in the proximal (^−/−^: 41.47 ± 2.35 cells, *n* = 6; ^+/−^: 32.30 ± 4.42 cells, *n* = 6; ^+/+^: 19.04 ± 2.74 cells, *n* = 6; Figure 3C) and distal colon (^−/−^: 38.47 ± 3.12 cells, *n* = 6; ^+/−^: 26.81 ± 5.65 cells, *n* = 6; ^+/+^: 20.8 ± 3.02 cells, *n* = 6; Figure 3C). *Shank3B^+/−^* mice also exhibited an increased number of enteric neurons per ganglion in the distal colon, but this did not correspond to an increase in myenteric plexus density (Figures 3A–C). No significant alterations were observed in the small intestine (Figures 3A–C, Supplementary Figure S2).

Enteric neurons regulate GI motility via innervation of GI smooth muscle, and both diarrhea and constipation are common among those with ASD (Bjørklund et al., 2020; Fung and Vanden Berghe, 2020). To assess global changes in GI motility, we gavaged mice with carmine dye and recorded the amount of time before mice defecated a dyed fecal pellet. *Shank3B^−/−^* and *Shank3B^+/−^* mice took longer to pass a dyed fecal pellet, indicative of slower whole-GI motility (^−/−^: 188.8 ± 17.5 min, *n* = 15; ^+/−^: 189.4 ± 17.3 min, *n* = 13; ^+/+^: 125.3 ± 8.8, *n* = 9; Figure 3D).

**Figure 4:**
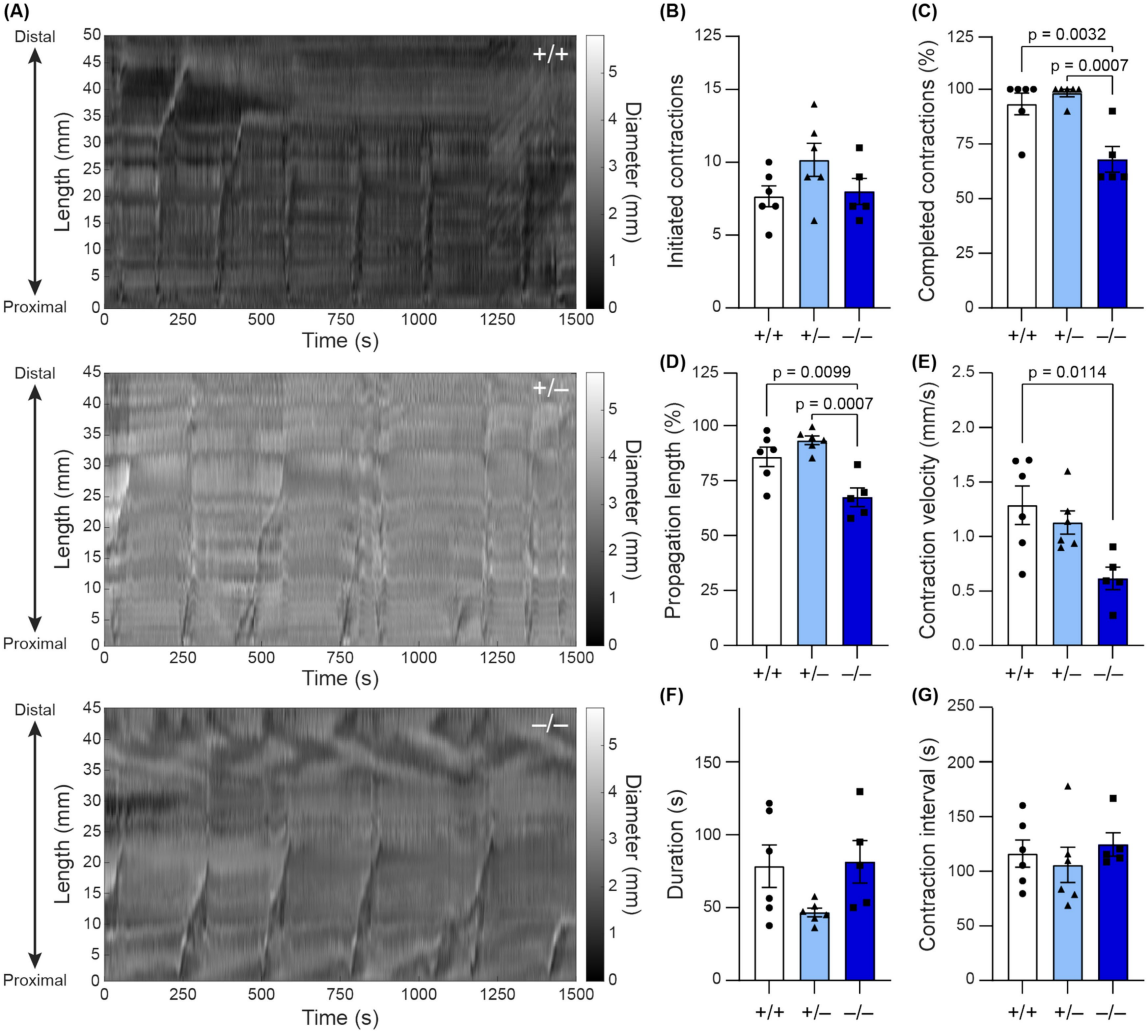
**(A)** Spatiotemporal maps track colonic contractions in *ex vivo* colonic preparation across time (x-axis) and at different parts of the colon (y-axis). The diameter of the colon is indicated by the grayscale value. Contractions appear as vertical stripes in the map. **(B–G)** Quantification of colonic contraction properties from spatiotemporal maps (*n* = 5–6). Data reported as mean ± SEM, with individual data points representing mean values from each animal. One-way ANOVA with Tukey’s multiple comparison test. **(B)** Number of initiated contractions. **(C)** Average percentage of colon involved in contraction. **(D)** Percentage of contractions that completed propagation along the entire colon (as defined by propagating > 80% of the colon). **(E)** Average contraction velocity. **(F)** Duration of contraction (how long the contraction lasts). **(G)** Interval between contractions.

While the carmine dye assay revealed that *Shank3* deletion leads to slowed whole-GI motility, it remained unclear whether this slowed motility stemmed from altered extrinsic innervation or differences in local ENS activity (Browning et al., 2017). Since our immunofluorescence staining revealed alterations in the organization of colonic myenteric plexus, we employed an *ex vivo* motility assay to further assess intrinsic colonic motility (Swaminathan et al., 2016). Using spatiotemporal maps, we characterized specific features of colonic moving contraction (CMC) events (Figure 4A). While the number of contractions initiated in the proximal colon was not significantly different across genotypes (^−/−^: 8 ± 2, *n* = 6; ^+/−^: 10.2 ± 2.8%, *n* = 6; ^+/+^: 7.7 ± 1.8, *n* = 5; Figure 4B), in *Shank3B^−/−^* mice, the average percentage of colon length that was involved in each CMC was lower (^−/−^: 68 ± 4.3%, *n* = 6; ^+/−^: 94 ± 2.0%, *n* = 6; ^+/+^: 86 ± 4.5%, *n* = 5; Figure 4C). Based on the average percentage of colon involvement in the CMCs of *Shank3B^+/+^* mice, CMCs were considered to reach the distal colon if they propagated >80% along the length of the colon. The number of CMCs that completed propagation to the distal colon was lower in *Shank3B^−/−^* mice (^−/−^: 68 ± 5.8%, *n* = 6; ^+/−^: 98 ± 1.7%, *n* = 6; ^+/+^: 93 ± 4.9%, *n* = 5; Figure 4D). Furthermore, individual CMC velocity was reduced in *Shank3B*^−/−^ and *Shank3B*^+/−^ mice, with *Shank3B^+/−^* mice showing a partial phenotype (^−/−^: 0.62 ± 0.10 mm/s, *n* = 6; ^+/−^: 1.13 ± 0.11 mm/s, *n* = 6; ^+/+^: 1.29 ± 0.17 mm/s, *n* = 5; Figure 4E). There were no significant differences in CMC duration or inter-CMC interval between *Shank3B^−/−^*, S*hank3B^+/–^*, and *Shank3B^+/+^* mice (Figures 4F,G).

## 4 Discussion

GI comorbidities are common among individuals with ASD, but their pathophysiology is not well understood (Bjørklund et al., 2020). In this study, we asked whether mutation of the ASD-linked gene *Shank3*, linked to Phelan-McDermid Syndrome (PMS) in humans, leads to alterations in ENS and GI tract structure and function that may manifest as GI comorbidities. In humans, mutations in one copy of *Shank3* lead to PMS; thus we examined whether GI functional differences are recapitulated in *Shank3B^+/−^* mice as well as *Shank3B^−/−^* mice. First, we found that SHANK3 expression was significantly decreased in *Shank3B^−/−^* mice. In heterozygous *Shank3B^+/−^* mice, SHANK3 expression was significantly decreased in the duodenum and proximal colon, but not in the distal colon, where expression trended lower, but was not statistically significant.

We found that knock out of SHANK3 Δex13-16 results in altered intestinal morphology not only in the duodenum but also in the colon. *Shank3B^−/−^* mice possessed shorter duodenal villi as well as shorter colons with thicker muscular walls. This knockout also increased paracellular permeability in small intestine mucosa. Myenteric plexus innervation was denser in the distal colon of *Shank3B^−/−^* mice, and enteric neuron count was higher. *Shank3B^−/−^* mice had longer whole-GI transit times. In *ex vivo* colon preparations, individual CMC velocity was slowed, and a lower proportion of CMCs involved the distal colon in *Shank3B^−/−^* mice. Despite these alterations in GI morphology and function, the weight of *Shank3B^−/−^* mice did not significantly differ from *Shank3B^+/+^* mice. To our knowledge, this study is the first to show that knockout of SHANK3 Δex13-16 results in slowed whole GI motility and disrupted CMC patterns, and that these alterations in GI motility are accompanied by ENS hyperplasia in the distal colon.

The severity of these alterations differed in a gene-copy dependent manner. In assays where *Shank3B^−/−^* mice were found to be significantly different from *Shank3B^+/+^* mice, heterozygous *Shank3B^+/−^* mice exhibited an intermediate but not always significant phenotype. *Shank3B^+/−^* mice exhibited a modestly but significantly reduced villi:crypt ratio, increased plexus density in the distal colon, and decreased whole-GI tract transit speed. The mild GI phenotypes in *Shank3B^+/−^* mice suggest an opportunity to use this model to identify environmental factors or biological pathways that exacerbate GI symptoms in mice with genetic vulnerability to ASD.

The results from the *in vivo* permeability assay, which evaluates mucosal permeability with extrinsic innervation to the mucosa still intact, corroborate previous histochemical markers of increased intestinal permeability in other *Shank3* KO models, *Shank3ΔC* KO (Δex21) (Wei et al., 2017) and *Shank3αβ* (Δex11) (Sauer et al., 2019). Although each *Shank3* KO model knocks out different exons, resulting in disruption of different protein domains, increased intestinal permeability is observed in each model. These convergent findings inspire future studies aimed at further characterization of tight junction structure and the proteins that interact with SHANK3 within intestinal epithelial cells.

scRNA-seq data of the ENS in mouse ileum and colon has also indicated that *Shank3* is expressed in subsets of excitatory and inhibitory motor neurons, as well as in some interneurons, secretomotor neurons, and sensory neurons (Drokhlyansky et al., 2020). Immunofluorescence data from this study corroborates immunohistochemical studies that identify SHANK3 expression in stomach myenteric neurons (Raab et al., 2010). Although the Shank3B ASD model globally knocks out most *Shank3* isoforms, results from the *ex vivo* motility assays indicate that loss of *Shank3* in intrinsic enteric neurons can alter motility even without the influence of extrinsic innervation. Notably, we found significant differences in ENS organization and CMC propagation in the distal colon, where scRNA-seq data has revealed higher expression of glutamate receptors compared to the rest of the GI tract (Drokhlyansky et al., 2020). However, whether the excitability of enteric neurons is altered in mice with *Shank3* mutations remains to be tested.

In the CNS, SHANK3 is well known as a scaffolding protein in the post-synaptic density of excitatory glutamatergic synapses. Loss of *Shank3* in the CNS leads to alterations in dendritic spine morphology, reduced glutamatergic synaptic transmission, and impaired synaptic plasticity (Bozdagi et al., 2010; Wang et al., 2011). In the periphery, *Shank3* mutation was shown to alter acetylcholine receptor clustering in myotubes at the neuromuscular junctions in individuals with PMS (Lutz et al., 2020). However, the molecular function of SHANK3 in ENS synapses and how it regulates specific neurotransmitter receptor expression is unknown. Future studies may investigate whether *Shank3* mutations result in altered distribution of cholinergic and glutamatergic varicosities in the ENS, particularly in relation to cell types that are common downstream targets of glutamatergic neurons, such as calretinin^+^ excitatory motor neurons and secretagogin^+^ sensory neurons (Hamnett et al., 2024). Whether SHANK3 expression varies across different subtypes of ENS neurons and synapses, or if SHANK3 mutation results in altered populations of enteric neuron subtypes, remains to be explored.

Nonetheless, glutamatergic signaling in the ENS has been shown to modulate myenteric neuron excitability via activation of mGluR channels on sensory interneurons and increase the force, but not frequency, of CMCs through activation of ENS AMPA receptors (Seifi and Swinny, 2016; Swaminathan et al., 2019). Furthermore, activation of VGLUT2 glutamatergic neurons led to an increase in colonic propulsion (Hamnett et al., 2024). Some enteric glutamatergic neurons have been shown to also produce acetylcholine— cholinergic neurons synchronously activate to facilitate motility (Tong et al., 2001; Filpa et al., 2016; Spencer et al., 2018). Taken together, these findings raise the possibility that *Shank3* mutation may decrease GI motility by altering the excitation of motility circuits via sensory interneurons, or by directly altering the synchronicity or strength of cholinergic signaling.

We find that mice with *Shank3* mutations exhibit alterations in GI tract and ENS morphology, which are accompanied by differences in intestinal barrier permeability and colonic motility. Further investigation will focus on identifying mechanisms underlying alterations in GI function associated with *Shank3* mutations. Furthermore, given that the ENS continues to develop after birth, in part due to signaling from the gut microbiota (Rao and Gershon, 2016), and that *Shank3* plays a role as a postnatal regulator of synaptic plasticity, it would be worthwhile to track altered GI tract and ENS development at multiple time points during the animal’s development (Monteiro and Feng, 2017). Our findings add to the body of evidence that ASD-linked mutations can also result in biological changes local to the GI tract, identifying a locus of dysfunction outside of the brain which may play a role in the pathogenesis of GI comorbidities in those with ASD.

## Data Availability

The original contributions presented in the study are included in the article/Supplementary material, further inquiries can be directed to the corresponding author.
