# Supplementary material for: Shank3 mutation manifests in abnormal gastrointestinal morphology and function in mice
Source: Front Neurosci. 2025 Apr 17;19:1552369. doi: 10.3389/fnins.2025.1552369 (PMC12043642; doi:10.3389/fnins.2025.1552369)
Supplement: Supplementary file 1 [file Data_Sheet_1.pdf]

Supplementary Information

Shank3B mutation manifests in abnormal gastrointestinal morphology and function in mice

Gari L. Eberly[1,2#], Marie Manthey[2,3,4#], Karen K.L. Pang[2,4,5], Heba Hussein[6], Emmanuel Vargas Paniagua[2], Scott Machen[2], Sara Maeve Klingensmith[7], and Polina Anikeeva[2,3,4,5,8]

[1] MIT-Harvard Graduate Program in Health Sciences and Technology

[2] K. Lisa Yang Brain-Body Center, Massachusetts Institute of Technology

[3] Research Laboratory of Electronics, Massachusetts Institute of Technology

[4] McGovern Institute for Brain Research, Massachusetts Institute of Technology

[5] Department of Brain and Cognitive Sciences, Massachusetts Institute of Technology

[6] Department of Electrical Engineering and Computer Science, Massachusetts Institute of Technology

[7] Department of Biology, Wellesley College.

[8] Department of Materials Science and Engineering, Massachusetts Institute of Technology

* All correspondence should be addressed to: P.A. (anikeeva@mit.edu)

# These authors contributed equally.

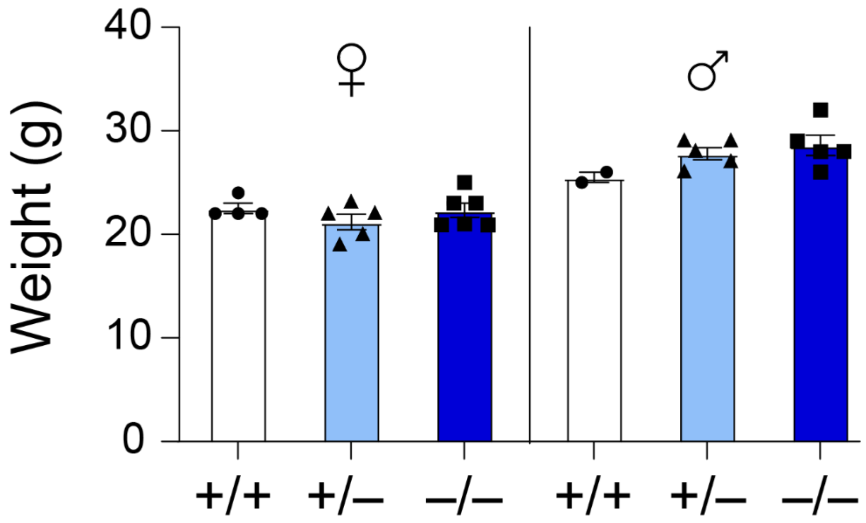

Supplementary Figure S1: Body weight is not significantly different between *Shank3B*$^{-/-}$, *Shank3B*$^{+/-}$, and *Shank3B*$^{+/+}$ mice.

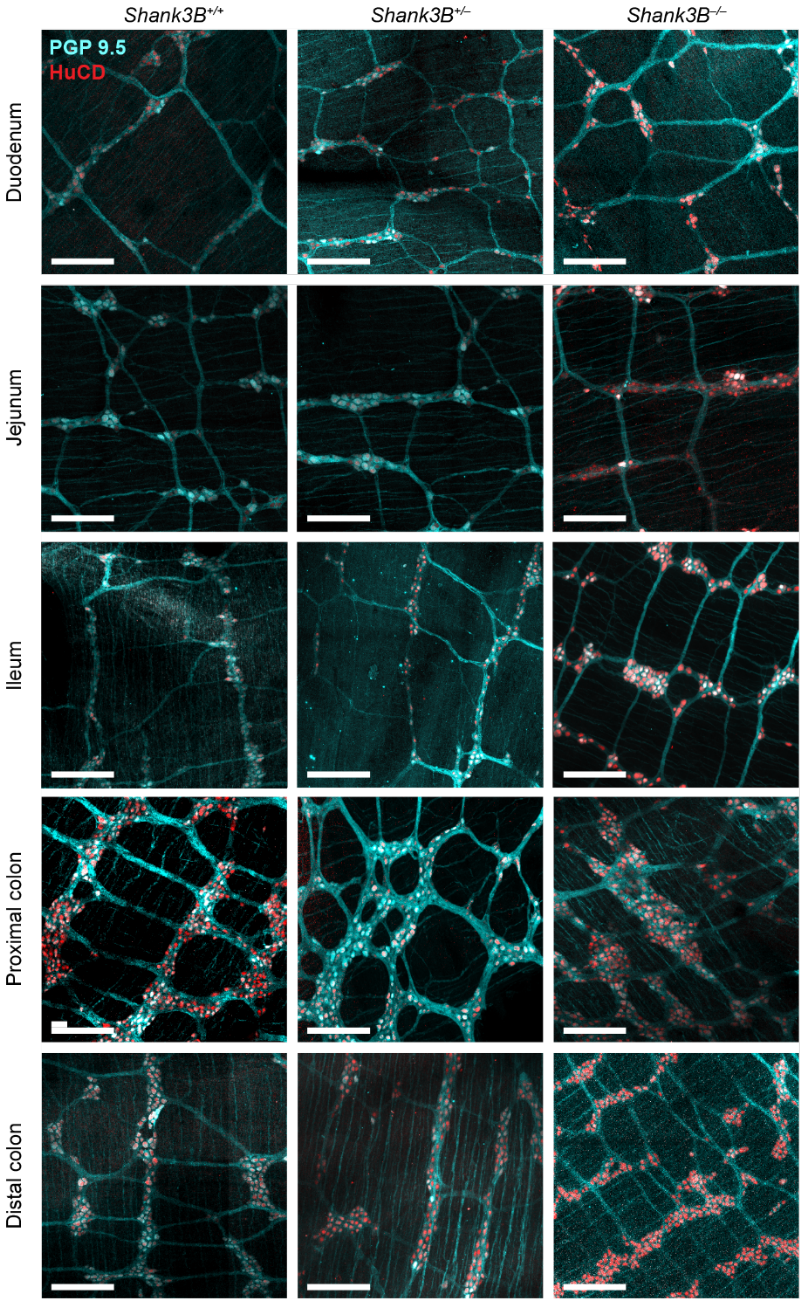

Figure S2: Representative images of myenteric plexus morphology in Shank3B$^{+/+}$, Shank3B$^{+/-}$, and Shank3B$^{-/-}$ mice. Scale bars indicate 200 µm. Neuronal bodies indicated by HuC/D (red) and projections indicated by PGP 9.5 (cyan).
